# Taurine Increases Zinc Preconditioning-Induced Prevention of Nitrosative Stress, Metabolic Alterations, and Motor Deficits in Young Rats following Intrauterine Ischemia

**DOI:** 10.1155/2021/6696538

**Published:** 2021-05-06

**Authors:** Alejandro Gonzalez-Vazquez, Ana-Karina Aguilar-Peralta, Constantino Tomas-Sanchez, Victor-Manuel Blanco-Alvarez, Daniel Martinez-Fong, Juan-Antonio Gonzalez-Barrios, Samuel Treviño, Lourdes Millán-Perez Peña, Victorino Alatriste, Guadalupe Soto-Rodriguez, Eduardo Brambila, Bertha Alicia Leon-Chavez

**Affiliations:** ^1^Facultad de Ciencias Químicas, Benemérita, Universidad Autónoma de Puebla, 14 sur y Av. San Claudio, Puebla, 72570 Puebla, Mexico; ^2^Facultad de enfermería, Benemérita Universidad Autónoma de Puebla, 27 sur 1304, Col. Volcanes, Puebla, 72410 Puebla, Mexico; ^3^Facultad de Medicina, Benemérita Universidad Autónoma de Puebla, 13 sur 2702, Col. Volcanes, Puebla, 72410 Puebla, Mexico; ^4^Departamento de Fisiología, Biofísica y Neurociencias, Centro de Investigación y de Estudios Avanzados del Instituto Politécnico Nacional, Apartado Postal 14-740, 07000 México, DF, Mexico; ^5^Laboratorio de Medicina Genómica, Hospital Regional 1° de Octubre, ISSSTE, Avenida, Instituto Politécnico Nacional #1669, 07760 México DF, Mexico; ^6^Centro de Química, ICUAP, Benemérita Universidad Autónoma de Puebla, 14 sur y Av. San Claudio, Puebla, 72570 Puebla, Mexico

## Abstract

Oxygen deprivation in newborns leads to hypoxic-ischemic encephalopathy, whose hallmarks are oxidative/nitrosative stress, energetic metabolism alterations, nutrient deficiency, and motor behavior disability. Zinc and taurine are known to protect against hypoxic-ischemic brain damage in adults and neonates. However, the combined effect of prophylactic zinc administration and therapeutic taurine treatment on intrauterine ischemia- (IUI-) induced cerebral damage remains unknown. The present work evaluated this issue in male pups subjected to transient IUI (10 min) at E17 and whose mothers received zinc from E1 to E16 and taurine from E17 to postnatal day 15 (PND15) via drinking water. We assessed motor alterations, nitrosative stress, lipid peroxidation, and the antioxidant system comprised of superoxide dismutase (SOD), catalase (CAT), and glutathione peroxidase (GPx). Enzymes of neuronal energetic pathways, such as aspartate aminotransferase (AST), alanine aminotransferase (ALT), and lactate dehydrogenase (LDH), were also evaluated. The hierarchization score of the protective effect of pharmacological strategies (HSPEPS) was used to select the most effective treatment. Compared with the IUI group, zinc, alone or combined with taurine, improved motor behavior and reduced nitrosative stress by increasing SOD, CAT, and GPx activities and decreasing the GSSG/GSH ratio in the cerebral cortex and hippocampus. Taurine alone increased the AST/ALT, LDH/ALT, and AST/LDH ratios in the cerebral cortex, showing improvement of the neural bioenergetics system. This result suggests that taurine improves pyruvate, lactate, and glutamate metabolism, thus decreasing IUI-caused cerebral damage and relieving motor behavior impairment. Our results showed that taurine alone or in combination with zinc provides neuroprotection in the IUI rat model.

## 1. Introduction

Hypoxic-ischemic encephalopathy (HIE) following intrauterine ischemia (IUI) is one of the leading causes of brain injury in neonates, and the effects can endure until adulthood [1, 2]. Approximately 15% to 20% of newborns affected with HIE die in the postnatal period, and 25% suffer from childhood disabilities such as epilepsy, visual and motor impairment, cerebral palsy, cognitive, and behavioral alterations [3]. The severity of these disabilities depends on the ischemia duration, damage expansion, and the affected brain region. The most common motor disabilities are muscular spasticity, reduced fine motor skills, akinesia, and walking discoordination [4].

In neonatal hypoxia-ischemia, an increase in reactive oxygen species (ROS) is known to occur in the brain at 96 h after a hypoxic-ischemic episode, to which the antioxidant system responds by increasing the activity of superoxide dismutase (SOD) and catalase (CAT) at postnatal day (PND) 11, which decreases afterward [5]. The decreased antioxidant activity of these enzymes and the reduced/oxidized glutathione (GSH/GSSG) ratio [6] lead to permanent high ROS levels at PND 45 [5]. Similar effects on the enzymatic activity of glutathione reductase (GR), glutathione peroxidase (GPx), and CAT have been reported in perinatal asphyxia [7]. These results collectively indicate the chronicity of oxidative stress and an inadequate antioxidant response after a cerebral hypoxia-ischemia event and have motivated the development of preventive and therapeutic approaches against oxidative stress.

IUI alters cerebral metabolism because it causes a deficiency of oxygen and nutrients essential for maturing the neurochemical circuitry in the neonatal phase, particularly the glutamatergic and GABAergic pathways in the cerebral cortex [8, 9]. Glucose is the primary energy source in the brain whose metabolism via the glycolysis pathway leads to pyruvate, which enters the Krebs cycle in the mitochondria or is converted into lactate by LDH [10, 11]. Faulty glucose metabolism impairs GABA synthesis from the glutamate source via a GABA shunt, a closed-loop independent glutamine process involving the Krebs cycle [12]. Through this cycle, *α*-ketoglutarate is transformed by the *α*-oxoglutarate transaminase into glutamate, which is decarboxylated to produce GABA. Moreover, energetic metabolism is favored by lactate through the activity of aspartate aminotransferase (AST), alanine aminotransferase (ALT), and lactate dehydrogenase (LDH) [13–15], which can also be incorporated into the glutamate, glutamine, and GABA cycles in neurons [16]. AST and ALT play an essential role in maintaining the energy metabolism in most tissues, including neonatal and adult cerebral tissue [17, 18]. Cytosolic AST increases glutamate levels, whereas mitochondria AST generates *α*-ketoglutarate, which maintains energy [19] and generates glutamine and the glutamate that ultimately generates GABA, which is decisive in maturing motor behavior and learning during postnatal development [20]. Cytosolic ALT is the primary isoform in astrocytes and neurons that, together with LDH, participates in energy metabolism and contributes to glutamate and pyruvate levels in physiologic and pathologic conditions [21, 22].

Hypoxia-ischemia also reduces the supply of nutrients such as glucose, glutamate, and N-acetyl-aspartate [23, 24] to the brain, which can cause cerebral injury. Placental dysfunction also contributes to micronutrient deficiencies in, e.g., copper, zinc, and manganese. Zinc deficiency is a frequent event in pregnant women (61%) [25]. This metal is an essential micronutrient involved in a great variety of cell functions, including antioxidant responses (binding to the sulfhydryl groups of biomolecules and metallothionein proteins) and stabilization of nitric oxide synthase (NOS) [26], SOD [27], and zinc finger proteins [28]. Accordingly, the supplementation of zinc (12 ppm) prevents cognitive and behavioral deficits following traumatic brain injury (TBI) [29], and antenatal zinc supplementation (16 ppm) improves memory consolidation in adult rats [30] and reduces neonatal sepsis and mortality rate in the offspring [31]. Studies on transient ischemia models in adult rats have shown that prophylactic zinc administration alone or in combination with selenium prevents nitrosative stress and increases chemokine and growth factor levels [32–34].

Preterm infants present reduced cystathionase activity, decreasing methionine conversion into cysteine and, finally, taurine production [35]. This metabolic deficiency of taurine is compensated by the maternal milk supply, which is known to contain taurine at high concentrations (45 to 50 mg/dL), thus avoiding deleterious consequences [36]. Nonetheless, ischemia-induced taurine deficiency during gestation [24] leads to growth retardation and impaired SNC development, which are manifested in adulthood as neurological dysfunction, reduced glucose tolerance, and vascular alterations [37]. Accordingly, taurine supplementation to neonates makes the cells resistant to ischemia-induced necrosis and apoptosis [38] during brain development [39].

Deleterious taurine effects have also been reported; for example, chronic supplementation with taurine causes a delay in auditory maturation [40]. However, the protective functions of taurine far exceed their deleterious effects. In neural tissues, taurine modulates intracellular calcium homeostasis [41]; increases antioxidative enzymes such as SOD, GPx [42], and GSH [43]; decreases glutamate-induced excitotoxicity [44, 45]; optimizes the growth and proliferation of human fetal brain cells; promotes neuron survival and neurite extensions [46]; reduces brain infarct volume; ameliorates morphological injury [42]; inhibits ischemia-induced apoptosis in neonatal rat cardiomyocytes [47]; downregulates caspase-3 expression [38]; and improves respiratory chain function after birth [48]. Additionally, maternal taurine supplementation partially prevents diabetes-induced oxidative stress in both mothers and embryos [49]. It also improves neural stem cell proliferation in rats with fetal growth restriction (FGR) by inhibiting Rho family factors [50].

Those antecedents sustain the hypothesis that supplementation of zinc or taurine, both individual and combined, can provide neuroprotection against transient IUI. On this basis, a 10 min IUI was performed on pregnant rats at embryonic day 17 (E17). Previously, zinc was administered to the mothers via drinking water from E1 to E16, followed by taurine treatment from E17 to postnatal day 15 (PND15). Motor behavior was evaluated in male pups at 5, 9, 11, 12, 13, and 14 PNDs using the motor test set reported previously [4]. The hierarchization score of the protective effect of pharmacological strategies (HSPEPS) was used to select the most effective treatment. The nitrosative stress was assessed through nitric oxide and lipid peroxidation in the temporoparietal cortex and hippocampus. The antioxidant activity of SOD, CAT, GPx, and GSH levels and the GSSG/GSH ratio were evaluated as a defensive mechanism against nitrosative stress. Additionally, energy metabolism through AST, ALT, and LDH activities was appraised as an index of cerebral function recovery. Our results showed that taurine blocked neuronal damage and increased the zinc preconditioning neuroprotection in the IUI rat model.

## 2. Materials and Methods

### 2.1. Experimental Animals

Pregnant female Sprague–Dawley rats (body weight 210 to 240 g) were obtained from the Care and Use of Laboratory Animals Unity of the Center for Research and Advanced Studies (CINVESTAV) and maintained in suitable rooms with the controlled conditions of temperature (22 ± 3°C) and light-dark cycles (12–12 h; light onset at 07 : 00). Rats were housed individually in Airlaw isolator polycarbonate cages (47.5 × 25.9 × 20.4 cm; Aller zone Lab products, Inc.; Seaford, DE, USA). Food (Laboratory Autoclavable Rodent Diet 5008, containing 73 ppm of zinc, 0.02% taurine, 0.43% methionine, and 0.35% cysteine) and drinking water were provided ad libitum. All procedures followed the current Mexican legislation, NOM-062-ZOO-1999 (SAGARPA), based on the Guide for the Care and Use of Laboratory Animals. The Institutional Animal Care and Use Committee approved the experimental procedures with the protocol number 0089-14 (CINVESTAV) and 3550 (Meritorious Autonomous University of Puebla, BUAP). All efforts were made to minimize animal suffering.

### 2.2. Supplementations

Pregnant rats were grouped into (1) Control w/t, without treatment and surgery; (2) IUI, with 10 min occlusion of the uterine arteries on E17; (3) zinc, with preconditioning administration from E1 to E16 of 30 ppm of ZnSO₄ heptahydrate (catalog # 221376; Sigma-Aldrich; San Luis, MO, USA) in drinking water equivalent to 12 ppm of atomic zinc concentration in the range of previous reports [30]; (4) Zn+IUI, with zinc preconditioning and IUI; (5) IUI+Tau, with therapeutic administration of 50 ppm of taurine (catalog # T8691; Sigma-Aldrich; San Luis, MO, USA) in drinking water from E17 to PND15 (the taurine concentration given corresponded to the taurine concentration in maternal milk [35]); and (6) Zn+IUI+Tau, with IUI and zinc preconditioning and taurine treatment.

### 2.3. Intrauterine Ischemia

Surgery was performed on pregnant rats anesthetized with ketamine (70 mg/kg) and xylazine (6 mg/kg) at E17 in sterile conditions using a biological safety cabinet class type A2 (Nuaire Laboratory Equipment; Plymouth, MN, USA). A 2 cm long medial laparotomy was made to expose the uterine horns, which were occluded with a clamp for 10 min (Bulldog Clamps, INS6000119; Kent Scientific Corporation; Torrington, CT, USA). The uterine horns were kept wet with a sterile phosphate-buffered solution (pH 7.4) (PBS), during surgery. Upon occlusion completion, the correct reperfusion of the arteries was visually verified. The uterus was replaced and washed with PBS containing sodium G penicillin (150 U), streptomycin (100 *μ*g/mL), and amphotericin B (25 *μ*g/mL) (Antibiotic-Antimycotic 100X; Gibco™; Thermo Fisher Scientific; Waltham, MA, USA) to prevent infection. The abdominal wall incision was sutured with absorbable nylon 3 (0) and the skin with silk 3 (0). Male pups were evaluated with a set of motor behavior tests over time (5, 7, 9, 11, 12, 13, and 14 PNDs) and euthanized on PND15 with ketamine (70 mg/kg) and xylazine (6 mg/kg) to obtain their brains.

### 2.4. Motor Behavior Tests

Only male pups were chronologically evaluated with a battery of motor tests described in detail elsewhere [4].

#### 2.4.1. Surface Righting (PND5)

The pups were placed on their backs on a bench pad and held in a supine position for 5 s. Afterward, they were released, and we recorded the time needed for them to return to the prone position (turning latency) for a minute. The test was performed three times per pup, and the average value was used to calculate the mean ± SEM of at least 5 pups per group.

#### 2.4.2. Cliff Aversion (PND9)

This test evaluates primary reflexes using the strength and coordination of the hindlimbs to avoid an abyss. PND9 pups were previously placed on a flat surface (31 × 24 × 17 cm) to explore the area for 30 s. Then, the head and forelimbs were placed on the box abyss, and the time to retreat from the abyss (retreatment latency) was measured. Unsuccessful trials were repeated with another pup.

The test was performed three times per pup, and the average value was used to calculate the mean ± SEM of at least 5 pups per group.

#### 2.4.3. Grip Strength (PND11)

PND11 pups were weighed and placed on a horizontal mesh (16 × 18 cm long × 1 mm^2^ thick) until the four limbs gripped the mesh. The mesh was then rotated slowly from a horizontal to a vertical position to defy the gravitational force. The falling time was measured (falling latency) to calculate the hanging impulse by multiplying the body weight by the falling latency. The test was performed three times per pup, and the average value was used to calculate the mean ± SEM of at least 5 pups per group.

#### 2.4.4. Forelimb Suspension (PND13)

PND13 pups are weighed and hung by the forelimbs on a wire crossing the top of a 3.5 L transparent plastic container with a cushioned floor. The falling latency was measured to calculate the hanging impulse by multiplying the body weight by the falling latency. The test was performed three times per pup, and the average value was used to calculate the mean ± SEM of at least 5 pups per group.

#### 2.4.5. Hindlimb Suspension (PND13)

PND13 pups were weighed and hung by the hind limbs on the border of a transparent glass container (15 cm × 6 cm) with smooth inner walls and a cushioned floor. The falling latency was measured to calculate the hanging impulse by multiplying the body weight by the falling latency. The test was performed three times per pup, and the average value was used to calculate the mean ± SEM of at least 5 pups per group.

#### 2.4.6. Negative Geotaxis (PND7, 9, and 12)

Pups of 7, 9, and 12 PNDs were placed upside down on a 45° tilt surface and held for 5 s. After releasing, the time to turn 180^o^ upward (turning latency) was measured during a 2 min trial. The test was performed three times per pup, and the average value was used to calculate the mean ± SEM of at least 5 pups per group.

#### 2.4.7. Hindlimb Foot Angle (PND14)

PND14 pups moved freely in an open field, and the angle of the two hind legs was video recorded during a 1 min gait. This angle depends on age and is useful in determining gait abnormalities. The angle was calculated with the ImageJ software (RRID:SCR_003070, National Institute of Health), and the average of 5 serial photographs per pup was obtained and used to calculate the mean ± SEM of at least 5 pups per group.

### 2.5. Biochemical Tests

The cerebral cortex and hippocampus of PND15 (*n* = 5 pups in each group) were individually dissected and mechanically homogenized in PBS. Afterward, the homogenates were centrifuged at 12,500 rpm for 30 min at 4°C with a Z216MK microcentrifuge (HERMLE Labortechnik GmbH; Wehingen, Germany). Aliquots of the supernatants were used to quantify nitrites, lipid peroxidation, GSH and GSSG levels, and enzymatic activities of AST, ALT, LDH, SOD, CAT, and GPx. Protein content was measured in the supernatants using the Coomassie blue method [32] to normalize the biochemical results.

### 2.6. Nitrite Quantification

Nitrite (NO_2_^−^) accumulation as NO production index was assessed in 10 *μ*L of supernatants using a colorimetrical reaction triggered by 10 *μ*L of Griess reagent, composed of equal volumes of 0.1% N-(1-naphthyl)ethylenediamine dihydrochloride and 1.32% sulfanilamide in 60% acetic acid [32]. Sample absorbances were measured at 540 nm with a NanoDrop 1000 Spectrophotometer (Thermo Fisher Scientific; Wilmington, DE, USA) and interpolated from a standard curve of NaNO_2_ (1 to 10 *μ*M) to calculate the nitrite concentration. The values were expressed as the *μ*M nitrite/mg protein.

### 2.7. Lipid Peroxidation Quantification

Malondialdehyde (MDA) and 4-hydroxyalkenal (4-HDA) concentration was measured (*n* = 5) as described previously [32]. The colorimetric reaction was triggered in 200 *μ*L of the supernatant after the addition of 650 *μ*L of 10.3 mM N-methyl-2phenyl-indole (Sigma-Aldrich; Saint Louis, MO, USA) diluted in a mixture of acetonitrile : methanol (3 : 1) and 150 *μ*L of methanesulfonic acid (Sigma-Aldrich; Saint Louis, MO, USA). The reaction mixture was vortexed and incubated at 45°C for 1 h and afterward centrifuged at 3000 rpm for 10 min. The absorbance was read at 586 nm in the supernatant with a SmartSpec 3000 spectrophotometer (Bio-Rad; Hercules, CA, USA). The absorbance values were compared to a standard curve of 0.25 to 5 *μ*M of 1,1,3,3-tetramethoxypropane (10 mM stock) to calculate the content of MDA and 4-HDA in the samples. The values were expressed as the nM MDA and 4-HDA/mg of protein.

### 2.8. CAT Enzymatic Activity

CAT enzymatic activity was quantified in 75 *μ*L of the supernatant in a spectrophotometer cuvette. After adding 330 *μ*L H_2_O_2_ (30 mM) and adjusting to 1 mL with PBS, the H_2_O_2_ absorbance change was continuously measured at 240 nm every 30 s with a spectrophotometer (Lambda EZ-150; PerkinElmer Company; Waltham, MA, USA). The results of enzymatic activity were reported as the U min^−1^/mg protein [51].

### 2.9. SOD Enzymatic Activity

Total SOD enzymatic activity in the supernatants was measured using pyrogallol as a substrate and recording its product at 420 nm, as reported in detail elsewhere [52]. In a quartz cuvette, 100 *μ*L of the supernatant was added and supplemented with 700 *μ*L of a Tris-HCl buffer solution, pH 8.2, and 50 *μ*L of EDTA. Subsequently, 50 *μ*L of the pyrogallol was added, and after 10 s of the reaction, the optical density (OD) changes were determined for one min at 420 nm with a spectrophotometer (Lambda EZ-150; PerkinElmer Company; Waltham, MA, USA) [52]. Upon achieving a 0.020 ± 0.001 absorbance change, the absorbance was continuously assessed for two more minutes. The results of enzymatic activity were reported as the U min^−1^/mg protein using the following equation:

(1) $$\begin{array}{c} \text{Enzymatic activity}=\left(\text{AV }\Delta \text{ODs}*100\text{ AV }\Delta \text{ODb}-100\right)*0.6, \end{array}$$

where AV is the average value, ΔODs is the sample OD difference, ΔODbis the blank OD difference from the reaction, and 0.6 is the constant factor.

Protein content was measured in the supernatants using the Coomassie blue method [32]. The results of enzymatic activity were reported as U min^−1^/mg protein.

### 2.10. GPx Enzymatic Activity

A Glutathione Peroxidase Kit (Item No. 703102) was used to measure GPx enzymatic activity following the manufacturer's instructions (Cayman Chemical Company; Ann Arbor, MI, USA). After perfusion with PBS, cerebral tissues were homogenized with 200 *μ*L of cold GPx sample buffer 10X (50 mM Tris-HCl, pH 7.5, 5 mM EDTA, and 1 mM DTT). In a 96-well plate, the background control wells contained 70 *μ*L of a GPx assay buffer (10X), 50 *μ*L of the cosubstrate mixture, and 50 *μ*L of NADPH. Positive control wells were supplemented with 50 *μ*L of an assay buffer, 50 *μ*L of a cosubstrate mixture, 50 *μ*L of NADPH, and 20 *μ*L of a diluted GPx Control. Subsequently, the background and positive control wells were read at 340 nm in triplicate, recording the NADPH absorbance change during each minute for 5 min. The sample wells contained 70 *μ*L of an assay buffer, 50 *μ*L of the cosubstrate mixture, 50 *μ*L of NADPH, and 20 *μ*L of a supernatant sample. The reaction was initiated by adding 20 *μ*L of cumene hydroperoxide to all wells. Finally, the absorbance at 340 nm was recorded every minute for 5 min using a microplate reader (Bechmark™, Bio-Rad; Hercules, California 94547, USA). The results of enzymatic activity were reported as NADPH nmol/min^−1^/mL.

### 2.11. Total Glutathione Assay (GSH + GSSG)

The quantification of the total glutathione levels was performed in a 96-well plate, as described elsewhere [53]. The samples were homogenized in a solution containing an equal proportion of 0.1% Triton-X (catalog # 9002-93-1 or X100; Sigma-Aldrich, St. Louis, MO, USA) and 0.6% 5-sulfosalicylic acid dihydrate (catalog # 247006; Sigma-Aldrich, St. Louis, MO, USA) and centrifuged at 8000 × g for 10 min at 2–4°C to obtain the supernatant. The background wells, in triplicate, contained 20 *μ*L of KPE (K_2_HPO_4_ buffer-EDTA, catalog # E9884; Sigma-Aldrich). The total glutathione was quantified in 20 *μ*L of supernatants by adding 60 *μ*L of the DNTB (5,5′-dithiobis (2-nitrobenzoic), catalog # D8113; Sigma-Aldrich, St. Louis, MO, USA) and 60 *μ*L glutathione reductase (catalog # G3664; Sigma-Aldrich, St. Louis, MO, USA). After 30 s of incubation, 60 *μ*L of *β*-NADPH (catalog # N1630; Sigma-Aldrich) was added to immediately read the 2-nitro-5-thiobenzoic acid formation at 412 nm every 30 s for 2 min using a microplate reader (Bechmark™, Bio-Rad, Hercules, California 94547, USA). The total glutathione concentration was determined via interpolation from a glutathione standard curve (26.4–0.4125 nM; catalog # PHR1359; Sigma-Aldrich, St. Louis, MO, USA). The results were expressed as *μ*M/mg of total proteins [53].

### 2.12. Oxidized Glutathione (GSSG)

GSSG quantification in 100 *μ*L of supernatants was performed by adding 2 *μ*L of 4-vinylpyridine (catalog # V3877; Sigma-Aldrich, St. Louis, MO, USA) and incubating the mixture for 1 h at room temperature. Subsequently, 6 *μ*L of triethanolamine (catalog # T58300; Sigma-Aldrich, St. Louis, MO, USA) was added to each sample, and the absorbance was read at 412 nm every 30 s for 2 min with a microplate reader (Bechmark™, Bio-Rad, Hercules, California 94547, USA). The concentration was calculated via interpolation from a GSSG standard curve ranging from 26.4 to 0.4125 nM (catalog # G4501; Sigma-Aldrich, St. Louis, MO, USA). The total GSSG concentration was expressed as *μ*M/mg of total proteins.

GSH concentration was the difference between total glutathione (GSH + GSSG) values and the oxidized glutathione (GSSG) [53].

### 2.13. Aspartate Aminotransferase (AST/GOT) Activity Assay

AST enzymatic activity was measured in 100 *μ*L of supernatant using the kit MIBEIS46-GOT(AST)-LQ following the manufacturer's instructions (Spinreact SAU; St. Esteve de Bas, Girona, Spain). We added 100 *μ*L of a 1 : 2 mixture of reagent R1 (Tris, pH 7.8 + lactate dehydrogenase (LDH) + malate dehydrogenase (MDH) + L‐aspartate) and reagent R2 (NADH + *α*‐ketoglutarate) at 25°C. Immediately, the NADH computation was continuously monitored at 340 nm for 3 min. Reference values reported in the datasheet are for male human adults for AST ranged up to 19 U/L at 25°C and 38 U/L at 37°C. The results of enzymatic activity were reported as U min^−1^/mg protein.

### 2.14. Alanine Aminotransferase (ALT/GPT) Activity Assay

ALT enzymatic activity was assessed in 100 *μ*L of the supernatant using the kit GPT_ALT_BEIS36_02-2011 following the manufacturer's instructions (Spinreact SAU; St. Esteve de Bas, Girona, Spain). After adding 100 *μ*L of a 1 : 2 mixture of reagent R1 (Tris, pH 7.8 + lactate dehydrogenase (LDH) + L‐alanine) and reagent R2 (NADH + *α*‐ketoglutarate), the absorbance was continuously monitored at 340 nm for 3 min at 25°C. Reference values reported in the datasheet for male human adults for ALT were up to 22 U/L at 25°C and 40 U/L at 37°C. The results of enzymatic activity were reported as U min^−1^/mg protein.

### 2.15. Lactate Dehydrogenase (LDH) Activity Assay

LDH enzymatic activity was assessed in 100 *μ*L of supernatant using the kit BEIS16_LDH_02-2015 following the manufacturer's instructions (Spinreact SAU; St. Esteve de Bas, Girona, Spain). After adding 100 *μ*L of a 1 : 2 mixture of reagent R1 (imidazole + pyruvate) and reagent R2 (NADH), the absorbance was monitored at 340 nm for 3 minutes at 25°C. The results of enzymatic activity were reported as U min^−1^/mg protein.

### 2.16. AST/ALT, LDH/ALT, and AST/LDH Ratios

The de Retis index (AST/ALT), a clinic prognostic reference of the tissue damage in several pathologies [54], was used to identify the glutamate metabolism direction. The AST/ALT ratio indicates if glutamate metabolism would be carried out in the mitochondria or cytosol based on the AST reaction (glutamate + oxaloacetate↔aspartate + *α*‐ketoglutarate). This reaction could be carried out preferentially in astrocytes participating in GABA synthesis and glutamate degradation [16]. Similarly, the AST/LDH ratio was considered an indicator of glutamate-pyruvate metabolism supplied by other molecular sources [55]. Based on the reactions of ALT (alanine + *α*‐ketoglutarate↔pyruvate + glutamate) and LDH (lactate ↔ pyruvate), we proposed that the LDH/ALT ratio indicates lactate-pyruvate metabolism [55]. The proposed LDH/ALT and AST/LDH ratios have no antecedents in the literature.

### 2.17. Statistical Analysis

All values were expressed as the mean ± SEM from at least 5 independent experiments. The biochemical results were analyzed with a one-way ANOVA using Dunnett's post hoc test to compare all groups with the Control w/t. The results of the motor behavior tests were analyzed with a Kruskal–Wallis one-way analysis of variance and a post hoc Dunn's test to compare multiple groups against the Control w/t, and a nonparametric Mann–Whitney *U* test was used to compare each experimental group with the IUI group. All statistical analyses were performed with the Prism software (GraphPad Prism; San Diego, CA, USA; RRID: SCR_0158070). *P* < 0.05 was considered to indicate statistical significance.

### 2.18. Hierarchization Score of the Protective Effect of Pharmacological Strategies (HSPEPS)

The values of motor behavior tests were expressed as the HSPEPS values, which indicate the efficacy of a pharmacological approach (register # MX2020010357). Motor dysfunction was given a value of zero, recovery was 1, and the improvement of the motor behavior above the Control was 2 (Table 1). HSPEPS was constructed with the sum of all performance scores of each treatment (Table 2).

## 3. Results

### 3.1. Motor Behavior Tests

**Figure 1:**
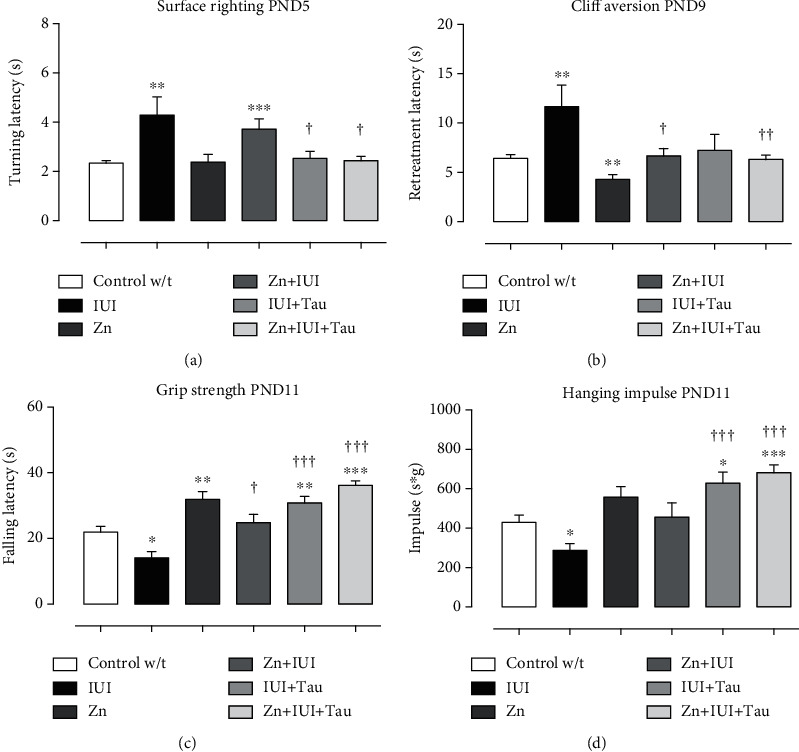
The effect of preconditioning zinc administration and therapeutic taurine treatment on intrauterine ischemia- (IUI-) induced alterations in surface righting, cliff aversion, grip strength, and hanging impulse. The values are the mean ± SEM (*n* = 5 to 8 pups). ^∗^*P* < 0.05, Kruskal–Wallis and post hoc Dunn's multiple comparisons test versus the Control w/t. ^†^When compared with the IUI, analyzed by Mann–Whitney *U* test. s: second; s∗g: second × grams.

The surface righting test in PND5 pups showed that the IUI increased the turning latency by 82.14 ± 44.17% (^∗∗^*P* = 0.01) compared with the Control w/t group. The preconditioning zinc administration did not change the turning latency (Zn+IUI), which was entirely prevented by taurine treatment alone (IUI+Tau) or combined with zinc (Zn+IUI+Tau) (Figure 1(a)).

The cliff aversion test among the PND9 pups showed that the IUI also increased the retreatment latency by 81.48 ± 33.84% (^∗∗^*P* = 0.0023) compared with the Control w/t group. The preconditioning zinc administration decreased the retreatment latency by 33.02 ± 7.29% (^∗∗^*P* = 0.0091) compared with the Control w/t group. All supplementations completely prevented an IUI-induced increase in retreatment latency (Figure 1(b)).

The grip strength test among the PND11 pups showed that the IUI decreased the falling latency by 30.25 ± 8.89% (^∗^*P* = 0.0113) compared with the Control w/t group. All treatments completely prevented the IUI-induced decrease in falling latency and increased it compared with the Control w/t group. The additional increase was 62.46 ± 16.64% (^†^*P* = 0.026) in the Zn+IUI group, 101.89 ± 12.82% (^†††^*P* < 0.0001) in the IUI+Tau group, and 136.6 ± 9.08% (^†††^*P* = 0.0001) in the Zn+IUI+Tau group compared with the IUI group. A significantly increased latency was observed in the Zn group (33.2 ± 10.72%, ^∗^*P* = 0.0023) compared with the Control w/t group (Figure 1(c)).

The hanging impulse test in PND11 showed that the IUI also decreased the hanging impulse by 33.2 ± 8.06% (^∗^*P* = 0.0172) compared with the Control w/t group. No statistical difference was found in the Zn group and Zn+IUI group compared with the Control w/t and IUI groups. Taurine alone or combined with zinc utterly prevented the IUI effect and additionally increased the hanging impulse compared with the Control w/t group by 46.12 ± 13.12% (^∗^*P* = 0.0169) in the IUI+Tau group and 58.55 ± 8.9% (^∗∗∗^*P* = 0.0004) in the Zn+IUI+Tau group (Figure 1(d)).

**Figure 2:**
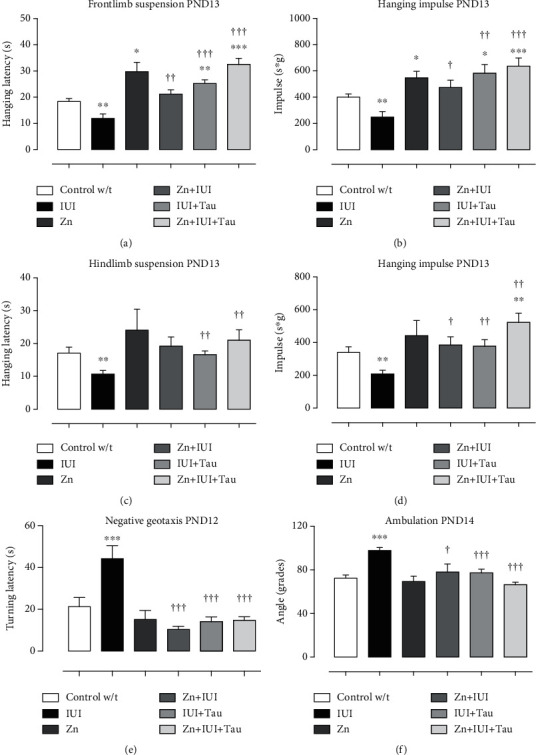
The effect of preconditioning zinc administration and therapeutic taurine treatment on intrauterine ischemia- (IUI-) induced alterations in hindlimb and forelimb suspension, negative geotaxis, and gait angle. The values are the mean ± SEM (*n* = 5 to 8 pups). ^∗^*P* < 0.05, Kruskal–Wallis and post hoc Dunn's multiple comparisons test versus the Control w/t. ^†^Mann–Whitney *U* test when compared with IUI. s: seconds; s∗g: seconds × grams.

The forelimb suspension test among the PND13 pups showed that IUI decreased the falling latency by 35.32 ± 9.52% (^∗∗^*P* = 0.0062) compared with the Control w/t group. Preconditioning zinc treatment prolonged the falling latency (62.36 ± 19.26%, ^∗^*P* = 0.0214) compared with the Control w/t and completely prevented IUI-induced effects. Taurine alone or combined with zinc prevented the IUI effect and additionally increased the falling latency by 37.42 ± 8.0% (^∗∗^*P* = 0.0026; IUI + Tau) and 77.5 ± 12.17% (^∗∗∗^*P* = 0.0001; Zn+IUI+Tau) compared with the Control w/t group (Figure 2(a)). When compared with the IUI group, the increase was 78.13 ± 14.06% (^††^*P* = 0.0037) in the Zn+IUI group, 112.48 ± 12.4% (^†††^*P* = 0.0001) in IUI+Tau group, and 174.42 ± 18.81% (^†††^*P* < 0.0001) in the Zn+IUI+Tau group (Figure 2(a)).

The hanging impulse of the forelimb suspension test showed a similar pattern to the falling latency in all groups. The hanging impulse decrease in the IUI group was 37.88 ± 10.2% (^∗∗^*P* = 0.0075). The other groups showed an increase in hanging impulse that was 36.88 ± 12.20% (^∗^*P* = 0.0214) in the Zn group, 45.62 ± 16.30% (^∗^*P* = 0.0426) in the IUI+Tau group, and 59.03 ± 15.42% (^∗∗∗^*P* = 0.0003) in the Zn+IUI+Tau group compared with the Control w/t group. Again, preconditioning zinc administration prevented the IUI-induced decrease in the hanging impulse (Figure 2(b)).

The hindlimb suspension test of the PND13 pups showed that IUI also decreased falling latency by 37.56 ± 6.9% (^∗∗^*P* = 0.0036) compared with the Control w/t group. The zinc group did not significantly modify the falling latency. Compared with the IUI group, the treatments of taurine alone (56.25 ± 10.35%, ^††^*P* = 0.0023) or combined with preconditioning zinc administration (97.57 ± 29.44%, ^††^*P* = 0.0072) prevented the IUI-induced decrease in falling latency (Figure 2(c)).

The hanging impulse measured by the hindlimb suspension test showed a similar pattern to the falling latency. The hanging impulse decrease in the IUI group was 46.34 ± 5.7% (^∗∗^*P* = 0.0059). This effect was prevented in the Zn+IUI group (84.51 ± 23.7%, ^†^*P* = 0.0165), IUI+Tau group (81.22 ± 19.23%, ^††^*P* = 0.0011), and Zn+IUI+Tau group (151.0 ± 26.54%, ^†††^*P* = 0.0012) compared with the IUI group. Taurine combined with zinc also increased the hanging impulse by 34.66 ± 14.23% (^∗^*P* = 0.0053) over the Control w/t group (Figure 2(d)).

The negative geotaxis test showed no differences in the turning latency among the groups until PND12. On this day, IUI increased the turning latency by 108.23 ± 29.24% (^∗^*P* = 0.026) compared with the Control w/t group. All treatments completely prevented the IUI-induced effect (Figure 2(e)).

The hindlimb angle test at PND14 showed that IUI increased the gait angle by 35.04 ± 4.07% (^∗∗∗^*P* = 0.0001) compared with the Control w/t group. All treatments completely prevented the IUI-induced effect. No effects were caused by preconditioning zinc administration (Figure 2(f)).

HSPEPS analysis faithfully showed the most effective treatment in preventing and improving IUI-induced motor disability (Table 3). The IUI group showed a minor score equal to 0, whereas all treatments had a total score equal to 10, indicating motor disability prevention. Scores higher than 10 indicated the ability to improve motor performance. Accordingly, a score = 10 was given for the preconditioning zinc administration, showing that this treatment completely prevented IUI-induced motor impairment, whereas the highest score was given to taurine alone (14) or taurine combined with zinc (15). Therefore, the best treatment was the preconditioning zinc administration combined with therapeutic taurine treatment because it improved motor ability better than the other treatments. Preconditioning zinc administration, therefore, improves the motor ability of healthy pups (Table 3).

### 3.2. Nitrites and Malondialdehyde and 4-Hydroxy-alkenals

**Figure 3:**
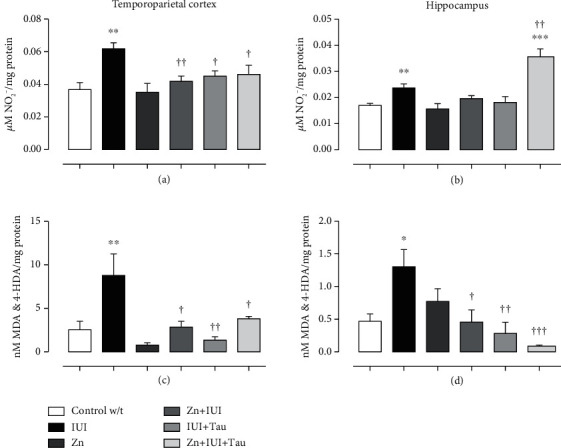
The supplementation of zinc alone or in combination with taurine decreased intrauterine ischemia-(IUI-) induced nitrosative stress. The values are the mean ± SEM (*n* = 5 pups). ^∗^*P* < 0.05 compared with the Control w/t group; ^†^*P* < 0.05 compared with the IUI group. One-way ANOVA and Dunnett's post hoc test.

Our results showed that IUI created a prooxidant environment in the temporoparietal cortex. Accordingly, IUI increased nitrite levels by 67.60 ± 10.03% (^∗∗^*P* = 0.0021) (Figure 3(a)) and MDA and 4-HDA levels by 247.28 ± 97.84% (^∗∗^*P* = 0.0066) (Figure 3(c)) compared with the Control w/t group. All treatments prevented the IUI-induced increase in nitrite (Figure 3(a)) and MDA and 4-HDA levels (Figure 3(c)) compared with the Control w/t group. In the hippocampus (Figure 3(b)), IUI increased nitrite levels by 39.34 ± 9.12% (^∗∗^*P* = 0.0048) and MDA and 4-HDA levels by 177.13 ± 55.73% (^∗^*P* = 0.0106) compared with the Control w/t group (Figure 3(d)). All treatments prevented the IUI-induced effect on nitrites and MDA and 4-HDA levels. Additionally, a significant 109.67 ± 17.48% (^∗∗∗^*P* = 0.0001) increase in nitrites was observed in the Zn+IUI+Tau groups compared with the Control w/t group (Figure 3(b)).

### 3.3. Antioxidant Activity

**Figure 4:**
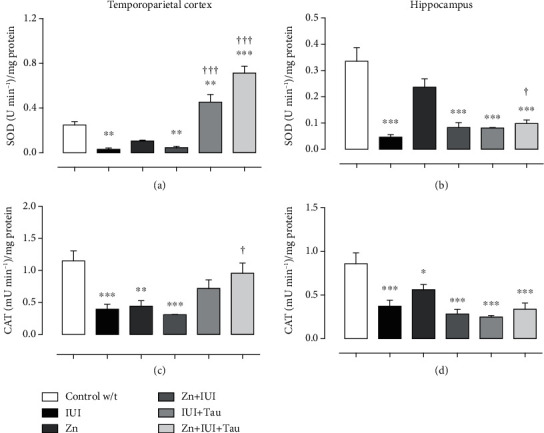
The effect of preconditioning zinc administration and therapeutic taurine treatment on the intrauterine ischemia-(IUI-) induced decrease in antioxidant activity. The values are the mean ± SEM (*n* = 5 pups). ^∗^*P* < 0.05 compared with the Control w/t; ^†^compared with IUI. One-way ANOVA and Dunnett's post hoc test.

In the temporoparietal cortex, IUI lowered SOD activity by 88.19 ± 5.65% (^∗∗^*P* = 0.0032) (Figure 4(a)). The preconditioning zinc administration did not modify this effect. In contrast, taurine alone or combined with zinc prevented the IUI effect and increased SOD activity compared with the Control w/t group. The increase was 1438.60 ± 233.32% (^†††^*P* = 0.0001) in the IUI+Tau group and 2324.93 ± 204.63% (^†††^*P* = 0.0001) in the Zn+IUI+Tau group compared with the Control w/t group. The zinc group showed no statistical difference compared with the Control w/t group (Figure 4(a)).

IUI also reduced CAT activity by 65.61 ± 6.8% (^∗∗∗^*P* = 0.0006). Only the preconditioning zinc administration combined with taurine treatment prevented the IUI-induced decrease in CAT activity (Figure 4(c)).

In the hippocampus, IUI decreased SOD activity (Figure 4(b)) by 86.20 ± 2.72% (^∗∗∗^*P* < 0.0001) and CAT activity (Figure 4(d)) by 56.67 ± 8% (^∗∗∗^*P* = 0.0004). All treatments failed to modify the IUI-induced decrease in SOD and CAT activities (Figures 4(b) and 4(d)). The preconditioning zinc administration decreased CAT activity by 34.65 ± 6.8% (^∗^*P* = 0.032) compared with the Control w/t group (Figure 4(d)).

**Figure 5:**
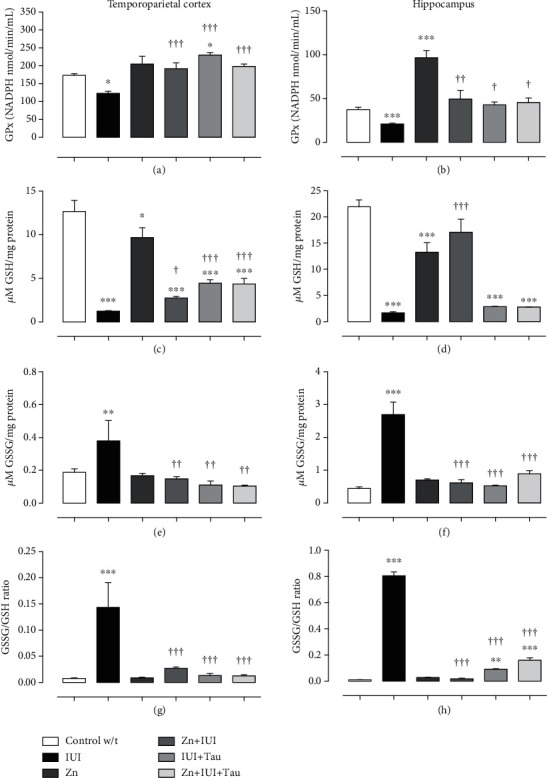
The preconditioning zinc administration and therapeutic taurine treatment increased the antioxidant ability of the GPx-glutathione system in intrauterine ischemia (IUI). The values are the mean ± SEM (*n* = 5 pups). ^∗^*P* < 0.05 compared with the Control w/t; ^†^compared with IUI. One-way ANOVA and Dunnett's post hoc test.

In the temporoparietal cortex, IUI decreased GPx activity (28.96 ± 3.2%; ^∗^*P* = 0.0287, Figure 5(a)) and GSH levels (98.23 ± 0.52%, ^∗∗∗^*P* < 0.0001, Figure 5(c)) and increased GSSG levels (102.35 ± 5.91%, ^∗∗^*P* = 0.008, Figure 5(e)) and the GSSG/GSH ratio compared to the Control group w/t. All individual or combined treatments entirely prevented the effects of IUI on GPx activity, GSSG levels, and the GSSG/GSH ratio (Figures 5(a), 5(e), and 5(g)) but only partially impacted GSH levels (Figure 5(c)). Compared with the IUI group, the increase in GSH levels was 123.45 ± 13.57% (^†^*P* = 0.0379) in Zn+IUI, 260.26 ± 32.13% (^†††^*P* < 0.0001) in IUI+Tau, and 252.59 ± 53.27% (^†††^*P* = 0.0001) in Zn+IUI+Tau (Figure 5(c)).

In the hippocampus, IUI reduced GPx activity by 43.89 ± 2.65% (^∗∗^*P* = 0.0004, Figure 5(b)) along with the GSH levels (92.39 ± 1.06%, ^∗∗∗^*P* < 0.0001, Figure 5(d)) but increased the GSSG levels (511.01 ± 86.99%, ^∗∗∗^*P* < 0.0001, Figure 5(f)) compared to the Control w/t. Again, all individual or combined treatments entirely prevented the IUI effect on GPx activity and GSSG levels (Figures 5(b) and 5(f)). Only Zn administration prevented a GSH-level decrease (Figure 5(d)) and a GSSH/GSH-ratio increase (Figure 5(h)). This latter effect was only partial in the treatments with individual taurine (88.70%) or combined with zinc (80.1%) compared with the Control w/t (Figure 5(h)).

### 3.4. Energy Metabolism Enzymes

**Figure 6:**
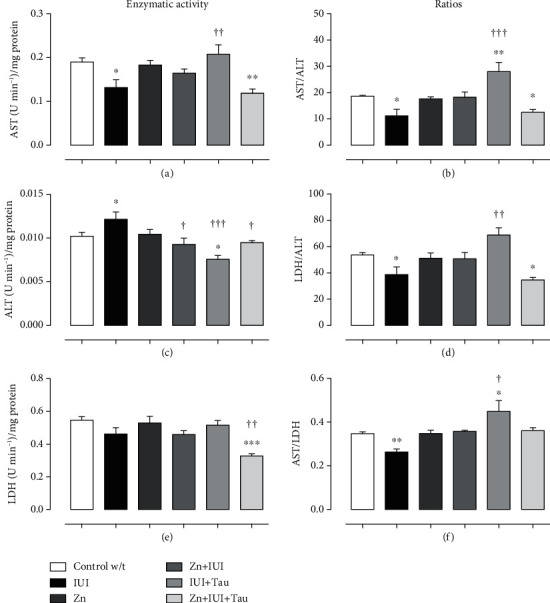
The combined supplementation of zinc and taurine recovers the intrauterine ischemia-(IUI-) induced effect on cellular metabolism in the temporoparietal cortex. The values are the mean ± SEM (*n* = 5). ^∗∗^*P* < 0.05 compared with the Control w/t; ^†^compared with IUI. One-way ANOVA and Dunnett's post hoc test.

In the temporoparietal cortex, IUI decreased the basal AST activity by 30.77 ± 9.4% (^∗^*P* = 0.0124) (Figure 6(a)) and increased the basal ALT activity by 19.85 ± 6.57% (^∗^*P* = 0.0251) (Figure 6(c)). The preconditioning zinc administration prevented only the IUI effect on ALT activity, whereas taurine treatment blocked the IUI-induced effect on both AST and AL (Figures 6(a) and 6(c)) and caused an additional decrease in ALT activity by 25.93 ± 4.33% (^∗∗^*P* = 0.0029) compared with the Control w/t. However, the combined administration of zinc and taurine did not change the IUI effect on AST but did block its effect on ALT activity (Figures 6(a) and 6(c)). The basal LDH activity was unaffected by IUI and treatments, although it was found to be decreased by 40.23 ± 2.75% (^∗∗∗^*P* = 0.0001) in the Zn+IUI+Tau group (Figure 6(e)). The preconditioning zinc administration did not modify the basal activity of the three enzymes studied (Figures 6(a), 6(c), and 6(e)).

Compared with the Control w/t group, IUI decreased the three studied ratios; namely, AST/ALT (Figure 6(b)) by 40.17 ± 10.27% (^∗^*P* = 0.0021), LDH/ALT (20.40 ± 3.85%, ^∗∗^*P* = 0.0042, Figure 6(d)), and AST/LDH (20.40 ± 3.85%, ^∗∗^*P* = 0.0011, Figure 6(f)). Only taurine administration significantly prevented the IUI induced effect on all ratios (Figures 6(b), 6(d), and 6(f)) and elicited an additional increase in AST/ALT ratio (64.04 ± 16.38%, ^∗∗∗^*P* = 0.0003, Figure 6(b)) and AST/LDH ratio by 29.29 ± 14.14% (^∗^*P* = 0.0376, Figure 6(d)) compared with the Control w/t group.

**Figure 7:**
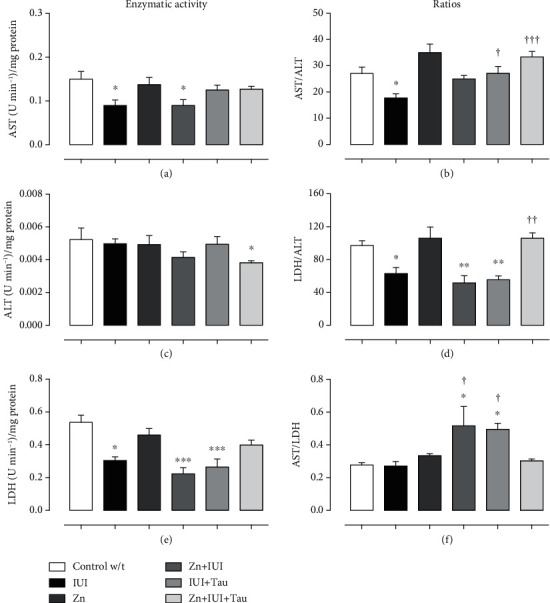
The combined supplementation of zinc and taurine recovered the intrauterine ischemia-(IUI-) induced effect on cellular metabolism in the hippocampus. The values are the mean ± SEM (*n* = 5). ^∗^*P* < 0.05 with when compared with the Control w/t; ^†^when compared with IUI (10 min). One-way ANOVA and Dunnett's post hoc test.

In the hippocampus, IUI decreased AST activity (Figure 7(a)) by 47.03 ± 4.76% (^∗∗∗^*P* = 0.0002) and LDH activity by 43.58 ± 4.10% (^∗∗∗^*P* = 0.0001) (Figure 7(e)) but did not affect ALT activity (Figure 7(c)). All treatments failed to prevent the IUI effect on AST and ALT. IUI also decreased the AST/ALT ratio by 34.48 ± 5.84% (^∗^*P* = 0.0412) and the LDH/ALT ratio by 35.02 ± 7.45% (^∗^*P* = 0.0204, Figure 7(d)) but did not change the AST/LDH ratio compared with the Control w/t group (Figures 7(b), 7(d), and 7(f)). The Zn preconditioning administration significantly prevented the IUI effect only on the AST/LDH ratio, which was additionally increased by 90.47 ± 43.41% (^†^*P* = 0.0409) compared with the Control w/t (Figure 7(f)). Similarly, therapeutic taurine administration blocked the IUI-induced decrease in the AST/ALT ratio (Figure 7(b)) and increased the AST/LDH ratio by 82.51 ± 13.76% (^†^*P* = 0.0498) (Figure 7(f)). Neither individual treatment (zinc or taurine) prevented the IUI effect on the LDH/ALT ratio (Figure 7(d)). However, the combined zinc and taurine administrations prevented the IUI effect on AST/ALT (Figure 7(b)) and LDH/ALT (Figure 7(d)).

## 4. Discussion

For the first time, this work reports an HSPEPS analysis and proves its usefulness to identify the most effective treatment for preventing behavioral disabilities in the HIE. HSPEPS analysis showed that taurine treatment individually or combined with prophylactic zinc administration surpassed the behavioral relief of the zinc preventive approach. The two taurine treatments recovered the reflexes of surface righting (PND5) and cliff aversion (PND9). They also increased the muscular force of the four limbs, as revealed by the tests of force grip strength (PND11), hanging impulse (PND11), and resistance force to suspension (PND13). Furthermore, they improved motor coordination and gait, as shown by the negative geotaxis (PND12) and hindlimb foot angle (PND14) tests. These behavioral enhancements were associated with biochemical improvements in the cerebral cortex and hippocampus at PND15—namely, recovering energy metabolism (AST and ALT enzymatic activity), improving antioxidant activity (SOD, CAT, GPx, and GSH), and decreasing lipid peroxidation.

The recovery of primary reflexes at PND5 elicited by taurine treatment individually or combined with the prophylactic zinc administration suggests that these treatments prevented the cellular damage caused by IUI in the dorsal gyrus of neonatal rats, as found in other IUI models [56, 57]. The stability of the gait and maintenance of balance and coordination resulting from increased muscle tone and grip force and a reduced hindlimb foot angle also suggest that this cellular gain extended to the brain regions involved in motor control [4]. These results collectively suggest that the treatments prevented the cell density reduction caused by oxidative stress in cortical, thalamic, and vestibular structures [58].

HSPEPS showed that zinc administration during the gestational period improved motor performance in healthy neonatal rats (score 14) and those subjected to IUI (score 10). This improvement could result from the positive zinc impact on embryonic neurogenesis [59, 60] and accurate CNS development in young rodents [60] and humans [61, 62]. Other studies also support the crucial role of zinc during development. Accordingly, it was shown that oral zinc supplementation reduces neonatal sepsis mortality and improves mental development [63], whereas zinc deficiency affects cognitive development and emotional behavior by decreasing neuronal activity [64, 65]. In contrast, other authors have reported no evidence that zinc supplements in children improve their motor or mental development [66]. However, zinc combined with iron promotes motor development and exploratory behavior in infants [67]. Zinc finger proteins could mediate the zinc-induced protection against ischemic damage at several levels. For instance, ZNF667 or Mipu1 regulates gene expression [68–70] activated by inflammatory mediators [71] and oxidative stress [72, 73]. Through this mechanism, zinc inhibits Bax [70] and Fas [72] expression, thus preventing apoptosis. The zinc-finger transcription factor Egr-1 (ZENK) has been shown to decrease the inflammatory process regulating IL-6-dependent JAK-STAT signaling [74], whereas ZFP580 modulates downstream ERK1/2 signaling with antiapoptotic roles in myocardial cells [75]. Moreover, zinc regulates epigenetic mechanisms acting as a cofactor of histone deacetylase (HDAC) [76].

HSPEPS analysis also showed that taurine, individually (score 14) or combined with zinc (score 15), corrected more behavioral deficits than only zinc administration, possibly by activating the additional protective mechanisms described in other approaches. Taurine participates in the differentiation of oligodendrocyte precursor cells (OPC) to produce myelin [77] and promotes different glutamate and cysteine metabolites [48] for the production of GSH in the mitochondria [43]. Taurine, acting as an agonist of the GABA receptors, can modulate electrophysiological activity [78] to protect against exercise-induced muscle injury [79, 80].

In this work, taurine supplementation of pregnant rats was not used as a control because this supplementation causes hyperexcitability and motor behavior deficits in neonates [81], modifying the GABAergic [82] and glutamatergic neurotransmission [83] critical for neurodevelopment [78, 84]. This deleterious taurine effect does not occur in old rats. Conversely, taurine prevents age-related declines in cognitive function by interacting with GABAergic neurotransmission [85], inhibiting glutamate excitotoxicity [45], and decreasing nitrosative stress [86].

Increased nitrosative stress and reduced antioxidant responses are typical in HIE. Accordingly, we found that IUI increased nitrosative stress (NO_2_^−^, MDA, and 4-HDA) in the temporoparietal cortex in the late phase (PND15). This increase in NO_2_^−^ could result from the activity of NO endothelial (eNOS) and neuronal (nNOS) synthase stimulated by IUI in the cerebral cortex at 24 h postreperfusion [87, 88], sustained by inducible iNOS activity up to PND8 and PND14 [89]. We also showed that the oxidative microenvironment resulting from the reduced antioxidant activity of SOD, CAT, GPx, and GSH levels and increased GSSG levels is still present at PD15, confirming previous findings in other perinatal asphyxia models [90, 91]. We also found that the decay of GPx antioxidant enzymatic activity endures up to PND15 after IUI, confirming that the immature brain has limited GPx activity, which makes it more susceptible to oxidative damage [92]. This susceptibility caused by the loss of antioxidant activity explains the sustained lipid peroxidation that we found at PND15.

In this work, the zinc concentration (12 ppm) was innocuous because it did not cause nitrosative stress and lipid peroxidation or change the basal antioxidant response in healthy brains at PND15. However, CAT activity was decreased, possibly by a posttranscriptional mechanism involving the deficient phosphorylation of ERK1/2 [93]. Moreover, GSH levels were decreased without modifying the GSSG/GSH ratio in the Zn group, suggesting that this decrease was due to the zinc-induced inhibition of glutathione reductase, as it occurs in astrocytes [94].

Conversely, prophylactic zinc administration prevented the IUI neurotoxic effect. This prevention results from the antioxidant ability of zinc acting as a cofactor of SOD (Cu-Zn), metallothionein, CAT, and GPx [95, 96] or as an inhibitor of NADPH oxidase [97] and NMDA [98]. Another mechanism may be a decrease in glutamate levels to avoid excitotoxicity and prevent the oxidative stress associated with ischemia/reperfusion [96]. Moreover, zinc increases GSH synthesis by stimulating glutamyl-cysteine ligase expression [96]. In agreement with this result, we also found a GSH increase in the Zn and Zn+IUI groups compared with the IUI group, which could account for the increased GPx activity in both the temporoparietal cortex and hippocampus at PND15 after IUI. Moreover, our result showing a decrease in the GSSG/GSH ratio in these cerebral regions following prophylactic zinc administration in the IUI reflects an antioxidative microenvironment, which explains the prevention of IUI-induced lipid peroxidation. The increase of the antioxidative microenvironment also explains the improvement of motor behavior in young rats after IUI. Together with the lack of a zinc effect on SOD and CAT activities, these results support the proposal that GPx1 plays a more critical role than SOD in defending against oxidative stress [99, 100].

In the temporoparietal cortex, taurine (50 ppm) blocked IUI-induced nitrosative stress by increasing SOD and GPx activities, according to previous findings, after cerebral hypoxic-ischemic injury in 7-day-old rats [42]. Moreover, taurine individually or combined with zinc also promoted an antioxidant microenvironment by increasing the GPx activity and decreasing the GSH/GSSG ratio. However, the combination of zinc and taurine showed a synergic effect only on SOD activity in the temporoparietal cortex, although it was the most effective treatment in improving motor behavior. The antioxidant action of taurine can result from its biotransformation into taurine chloramine and taurine haloamine via microglia/macrophages that decrease neuroinflammation, autophagy, ER stress, and apoptosis via the Nrf2 factor [101] in a hypoxic-ischemic process [44]. Furthermore, zinc and taurine chloramine are known to inhibit NO production in activated microglial cells in adult rats subjected to cerebral ischemia [102]. Antioxidant taurine action can also be potentiated by the endogenous taurine release stimulated by NO [103]. This mechanism could explain the NO increase and lipid peroxide reduction in the hippocampus in the Zn+IUI+Tau group. Taurine can also act through independent antioxidant mechanisms in the hippocampus, where it plays a critical nutritional role in neuronal cell growth, differentiation, and development. Moreover, taurine plays a crucial role in protein synthesis and, in the mitochondria, improves electron transport chain (ETC) activity and neural bioenergetics, thus avoiding excess ROS [86]. At the cell level, taurine primarily regulates cell volume as an osmoregulatory factor, attenuates electrical signals, and hyperpolarizes neurons by increasing the influx of chloride ions through three target receptors: GABA, glycine, and NMDA [78, 104].

The combination of preconditioning zinc administration with taurine treatment provided the best neuroprotection because it recovered motor activity (the highest HSPEPS), decreased nitrosative stress, prevented lipid peroxidation, and improved the antioxidant response. These results agree with those showing that taurine protects against absolute ethanol-induced gastric lesions by enhancing antioxidant activity and endogenous PGE2 production and attenuating NO production [105, 106]. However, more experiments are needed to identify the molecular mechanism underpinning the neuroprotective effect of prophylactic zinc administration and therapeutic taurine treatment in IUI.

The CK and CK-BB isoforms of brain creatine kinase have been considered diagnostic/prognostic biomarkers in serum and cerebrospinal fluid in neonatal HIE [107] together with S100, AST, and LDH [108, 109]. Increased CK-BB levels were previously found in all patients with stroke upon emergency service admission and remained high upon therapy termination [110], unlike AST and LDH, which decrease after recovery from stroke. There are no reports in the literature about zinc and taurine's effects on CK-BB in hypoxia-ischemia models to the best of our knowledge. It would be interesting to include CK and CK-BB markers in future studies to reinforce the effectiveness of the treatments against IUI-induced cerebral damage.

AST and ALT activity are markers of energy metabolism in the brain in physiological and pathological conditions. IUI is known to reduce energy metabolism in the brain, altering glucose metabolism as the primary energy source, modifying pyruvate metabolism in the Krebs cycle in mitochondria, and increasing lactate via the LDH [10, 11]. The affected neurons require lactate produced by the astrocytes to maintain the pyruvate levels and neural energy. To compensate for IUI-induced energy metabolism deficits, the transaminases (ALT and AST) increase glutamate and *α*-ketoglutarate levels [111]. Therefore, a decrease in ALT and AST alters the glutamine and glutamate sources from the metabolism and consequently alters the GABA synthesis in the astrocytes, which are critical for motor skills and learning in postnatal development [17, 18]. Moreover, ALT plays an essential role in development because it participates in aspartate and glutamate metabolism, which is critical for the maturation of glutamatergic structures in the second postnatal week [9, 112, 113]. The ratio of these enzymes indicates metabolic flux. A high AST/LDH ratio shows glutamate generation, while a low value reflects lactate production. An increased LDH/ALT ratio suggests lactate accumulation, whereas a decreased value indicates high pyruvate production. A high AST/ALT ratio also suggests glutamate production, while a decrease indicates pyruvate production preference.

Our results on the metabolic activity ratios suggest that the cerebral cortex needs pyruvate and lactate, which are related to cell proliferation and reactive astrogliosis, in response to IUI-induced cerebral damage [112, 114]. Our results also show that prophylactic zinc supplementation to healthy pregnant rats did not modify the physiological metabolism in the temporoparietal cortex and hippocampus. In contrast, it restored enzymatic activity in those regions in the IUI model. After IUI, taurine treatment promoted glutamate and lactate metabolic flux, suggesting that taurine favors cerebral cortex maturation via the glutamine-glutamate-GABA cycle [115–119]. Glutamate consumption favoring GABA synthesis leads to the prevention of excitotoxicity-induced neurodegeneration, which accelerates neuromotor recovery from IUI and improves cognition [120–122]. Moreover, lactate produced from glucose or glycogen in astrocytes is transferred to neurons that oxidize it into pyruvate to ensure an adequate energy supply [123]. On this basis, we suggest that the combination of zinc and taurine (Zn+IUI+Tau group) increased lactate and pyruvate flux between the astrocytes and neurons to modulate neuronal functions, such as excitability, plasticity, memory consolidation, and motor activity [123–125].

In the hippocampus, our results suggest that IUI favors pyruvate metabolism on PND15, indicative of mitochondrial metabolic recovery [126], which was not sufficient to restore the antioxidant system and motor ability. Prophylactic zinc administration did not improve AST and LDH enzymatic activity but favored glutamate and pyruvate metabolic flux. Taurine recovered AST and ALT activities and promoted a cytosolic flux of pyruvate and glutamate, which has been linked to processes of neuronal maturation and synaptic transmission in neurons *de novo* [127–129] in the dendritic layers, such as Schaffer's collaterals, stratum oriens, and stratum radiatum [130]. Likewise, the combined administration of zinc and taurine restored the enzymatic ratio to the control levels, suggesting that the establishment of lactate and glutamate physiological regulation in astrocytes and neurons [131] decreases IUI-caused excitotoxicity [132].

## 5. Conclusions

In summary, our findings demonstrate that IUI generates severe motor alterations resulting from multiple biochemical alterations in the young brain until PND15. The temporoparietal cortex and hippocampus were deeply affected by nitrosative stress and the depletion of antioxidant and metabolic enzymes. The prophylactic administration of zinc and taurine treatment individually or combined with zinc could prevent damage in these cerebral regions and, consequently, motor disability. However, these treatments exerted a region-dependent effect on energy metabolism, pyruvate-lactate, and glutamine-glutamate and GABA cycles, acting as a compensatory mechanism against ischemic damage.

HSPEPS showed that prophylactic zinc administration is effective against IUI, but taurine administration individually or combined with zinc provided an enhanced neuroprotector effect. A zinc-taurine combination improves motor activity and protects the temporoparietal cortex and hippocampus from metabolic fluxes of lactate and pyruvate, as indicated by our results at PND15. Therefore, taurine-based treatments could be used to prevent neuromotor damage in IUI.

## Figures and Tables

**Table 1:** Performance score for each treatment per animal.

Score	Performance
0	Lower than control
1	Equal to control
2	Better than control

**Table 2:** Hierarchization score of the protective effect of pharmacological strategies (HSPEPS).

Performance score	Recovering ratio
0 to 5	Inefficient
6 to <10	Moderate
=10	Efficient
>10	Enhanced

**Table 3:** Hierarchization score of the protective effect of pharmacological strategies (HSPEPS) in the motor recovery.

PND	Test	IUI	Zn	Zn + IUI	IUI + Tau	Zn + IUI + Tau
5	Surface righting	0	1	1	1	1
9	Cliff aversion	0	2	1	1	1
11	Grip strength	0	2	1	2	2
11	Hanging impulse	0	2	1	2	2
12	Geotaxis negative	0	1	1	1	1
13	Front-limb suspension	0	2	1	2	2
13	Hanging impulse	0	2	1	2	2
13	Hindlimb suspension	0	1	1	1	1
13	Hanging impulse	0	1	1	1	2
14	Hindlimb angle	0	1	1	1	1
	Total	0	14	10	14	15

## Data Availability

The data utilized to support the findings of this study are available from the corresponding author upon request.
